# Brain-Derived Neurotrophic Factor (BDNF) and Translocator Protein (TSPO) as Diagnostic Biomarkers for Acute Ischemic Stroke

**DOI:** 10.3390/diagnostics13132298

**Published:** 2023-07-06

**Authors:** Mayuri N. Tuwar, Wei-Hung Chen, Arthur M. Chiwaya, Hsu-Ling Yeh, Minh H. Nguyen, Chyi-Huey Bai

**Affiliations:** 1 School of Public Health, College of Public Health, Taipei Medical University, Taipei 106236, Taiwan; mayutuwar29@gmail.com; 2 Department of Neurology, Shin Kong Wu Ho-Su Memorial Hospital, Taipei 111045, Taiwan; m000735@ms.skh.org.tw (W.-H.C.); mirage.yeh@gmail.com (H.-L.Y.); 3 CLIME Group, Department of Biomedical Sciences, Division of Molecular Biology and Human Genetics, FMHS, Stellenbosch University, Francie Van Zijl Drive, Tygerberg, Cape Town 7505, South Africa; achiwaya@gmail.com; 4 School of Preventive Medicine and Public Health, Hanoi Medical University, Hanoi 100000, Vietnam; drminh.ttytlc@gmail.com; 5 School of Medicine, College of Medicine, Taipei Medical University, Taipei 106236, Taiwan

**Keywords:** acute ischemic stroke, brain-derived neurotrophic factor (BDNF), translocator protein (TSPO)

## Abstract

Brain-derived neurotrophic factor (BDNF) interacts with tropomyosin-related kinase B (TrkB) to promote neuronal growth, survival, differentiation, neurotransmitter release, and synaptic plasticity. The translocator protein (TSPO) is known to be found in arterial plaques, which are a symptom of atherosclerosis and a contributory cause of ischemic stroke. This study aims to determine the diagnostic accuracy of plasma BDNF and TSPO levels in discriminating new-onset acute ischemic stroke (AIS) patients from individuals without acute ischemic stroke. A total of 90 AIS patients (61% male, with a mean age of 67.7 ± 12.88) were recruited consecutively in a stroke unit, and each patient was paired with two age- and gender-matched controls. The sensitivity, specificity, and area of the curve between high plasma BDNF and TSPO and having AIS was determined using receiver operating characteristic curves. Furthermore, compared to the controls, AIS patients exhibited significantly higher levels of BDNF and TSPO, blood pressure, HbA1c, and white blood cells, as well as higher creatinine levels. The plasma levels of BDNF and TSPO can significantly discriminate AIS patients from healthy individuals (AUC 0.76 and 0.89, respectively). However, combining the two biomarkers provided little improvement in AUC (0.90). It may be possible to use elevated levels of TSPO as a diagnostic biomarker in patients with acute ischemic stroke upon admission.

## 1. Introduction

Strokes are the world’s second leading cause of disability and death, and it has a serious impact on both individuals and society [1]. In addition, it is thought to be an age-related condition, with both modifiable and nonmodifiable risk factors. Studies have indicated that gender is highly associated with stroke, in that young women tend to have a higher or equal risk of stroke than men. However, men tend to have a higher risk at an advanced age. In addition, racial factors significantly contribute to the development of stroke, and this is evident from the fact that African Americans have double the risk when compared to Caucasians [2]. Compared to Western populations, Chinese people have a greater stroke incidence and mortality rate, as well as a higher level of disability-adjusted life years [3]. Strokes come in different forms, such as: subarachnoid hemorrhage, with a frequency of around 5%; intracerebral hemorrhage, with 14%; and a majority of cases being ischemic stroke, constituting 80% of cases [4,5,6].

Brain-derived neurotrophic factor (BDNF) is a neurotrophin, and is crucial for the differentiation, development, growth, and survival of central nervous system cells [7,8]. BDNF interacts with tropomyosin-related kinase B (TrkB) to promote neuronal growth, survival, differentiation, neurotransmitter release, and synaptic plasticity [7,9]. It has been shown that BDNF has a significant role in treating acute brain injuries as a result of its great abundance in the cerebral cortex, as well as its ability to decrease neuronal damage and heal brain damage. The use of BDNF or its mimics has shown encouraging results in preclinical studies, and clinical trials are expected to begin using these therapies shortly [10]. Changes in the levels of BDNF, both in the CNS tissues and the circulating blood, have been linked to the etiology of neurodegenerative diseases, such as Alzheimer’s disease (AD), multiple sclerosis (MS), Huntington’s disease (HD), amyotrophic lateral sclerosis (ALS), Parkinson’s disease (PD), and ischemic stroke (IS) [11,12,13].

Translocator protein (TSPO) refers to an external membrane protein involved in the regulation of steroidogenesis, and it is usually found in specific types of tissue or brain [14,15,16]. TSPO is associated with certain physiological and cellular functions, including cellular respiration and metabolism [17,18]. Studies have indicated that inflammation of CNS is associated with the elevation of TSPO in activated macrophages, microglia, and reactive astrocytes [19]. Drawing from these properties, it is evident that TSPO forms part of a crucial marker for the definitive diagnosis and development of various psychiatric and neurological conditions, such as depression, traumatic brain injury, schizophrenia, stroke, neurological disorders, and anxiety [20]. The most recent research regarding TSPO involves the use of imaging methods to identify inflammatory brain cells. Through marking PK-11195, a high-affinity and selective TSPO ligand with various radioisotopes, the distribution of TSPO can be calculated and visualized through the application of such imaging techniques as single-photon emission computerized tomography (SPECT) or positron emission tomography (PET) [21].

Several studies have compared the plasma levels of BDNF in individuals presenting with and without ischemic stroke [22,23,24]. A few studies also discovered that BDNF and TSPO can be utilized as diagnostic markers for acute ischemic stroke [25,26].

In this study, we determined the diagnostic accuracy of plasma BDNF and TSPO levels in discriminating new-onset acute ischemic stroke (AIS) patients from individuals without acute ischemic stroke.

## 2. Materials and Methods

### 2.1. Study Participants

In this study, 90 IS patients (within 48 h of stroke onset) were consecutively recruited in a stroke unit from July 2014 to July 2015, and again from November 2017 to September 2019. Every case was paired with two community-based controls that were age and gender-matched. The 180 community-based controls, aged 40 to 85 years, were from one wave (2016) of a prospective cohort study (2004 to now) in the communities around the hospital. 

Patients were eligible for inclusion as cases if they did not show any history of psychiatric or neurological disorders such as HD, stroke, transient ischemic attack (TIA), MS, PD, AD, and ALS. In addition, patients were eligible if they had suffered their first ever AIS according to the neurologist’s clinical definition, as well as if they had been admitted to the emergency department or stroke unit within 48 h after the onset of symptoms. Moreover, the exclusion criteria involved those with infection, those without complete baseline data, and cases where there was no match with the control group based on age (±5 years) and gender.

Those participants were eligible for inclusion if they were healthy volunteers without any history of such neurodegenerative conditions as stroke, TIA, or PD, among others. After exclusion, 90 patients with AIS were included, and all of the patients were followed up to the point of being discharged.

### 2.2. Study Ethics

The study was approved by the Investigational Review Board of Shin Kong Wu Ho-Su Memorial Hospital (IRB no. 20170701R) and conformed with the principles of the Declaration of Helsinki. All of the participants’ written informed consent was received before they participated in the study.

### 2.3. Data Collection

In patients with known stroke, information was gathered from the medical charts of patients, in addition to standardized structured questionnaire interviews, which were conducted by expert interviewers. The structured questionnaire involved information about admission demographics and the findings of radiographical, clinical, and laboratory evaluation. One study-certified neurologist took charge of stroke diagnoses and neurological status, while a trained certified nurse was given the responsibility of assessing the functional outcome of the patients. The stroke diagnosis was verified by a stroke neurologist based on symptoms and brain imaging, either computer tomography (CT) or magnetic resonance imaging (MRI). Ischemic strokes were divided into five subgroups based on the following Trial of ORG 10,172 in Acute Stroke Treatment (TOAST) criteria: large artery atherosclerosis, small vessel occlusion, cardio-embolism, specific pathogenesis, and undetermined pathogenesis. The National Institutes of Health Stroke Scale (NIHSS) was used to assess neurological status at admission. The severity of stroke was classified as moderate stroke (5–15), moderate to severe (16–20), and severe stroke (≥21) using the NIHSS. Functional outcomes at discharge were assessed using the modified Rankin scale (mRS).

Furthermore, study information was applied so as to help gather information from community-based participants through a standardized structured questionnaire and physical examination, which were all performed by well-trained interviewers. This process was used in order to collect such information as the patient’s demographic characteristics, smoking status, the onset time of stroke symptoms, and current medication for hypertension (HTN) or diabetes mellitus (DM), as well as their family history of these diseases.

### 2.4. Variable Definitions

The definition of HTN involved a systolic blood pressure (SBP) level equal to or greater than 140 mmHg, a diastolic blood pressure (DBP) level equal to or greater than 90 mmHg, and a physician’s diagnosis of HTN. In contrast, the definition of DM involved a fasting blood glucose level equal to or greater than 126 mg/dL, as well as glycosylated hemoglobin (HbA1c) ≥ 6.5%. Hyperlipidemia was defined as a total cholesterol (TCHO) ≥ 200 mg/dL, low-density lipoprotein (LDL) > 130 mg/dL, and triglycerides (TG) ≥ 150 mg/dL.

Furthermore, all known stroke patients were subjected to follow-up to the point of death or discharge. In addition, techniques such as brain MRI, CT scan, or both were undertaken to define and assess the size and site of the brain infarct lesion. On the same note, the stroke severity was classified upon discharge as moderate or minor if <25 on the NIHSS, and severe if ≥25. In addition, mRS assessed functional outcomes at discharge, for which an mRS score < 3 reflected a favorable outcome.

### 2.5. Blood Collection

Participants’ blood samples were collected upon admission to the emergency department if the emergency physician or neurologist diagnosed a suspected stroke at that time. The date and hour of admission, blood draw, and medical procedures were recorded in enrollment documentation. The sampling time length was identified as the time interval in hours between admission and blood draw. Samples of fasting blood were obtained and centrifuged at 3000× *g* for 15 min within 2 h of collection, and at room temperature. Thereafter, they were separated into different tubes containing plasma, serum, and buffy coats and stored at −80 °C until analysis. Levels of hemoglobin (Hb), hematocrit (HCT), platelets, white blood cells (WBC), glutamic oxaloacetic acid transaminase (GOT), fasting blood glucose, HbA1c, blood urea nitrogen (BUN), creatinine, high-sensitive C-reactive protein (Hs-CRP), high-density lipoprotein (HDL), TCHO, and LDL were also assessed in the lab.

### 2.6. TSPO and BDNF Measurement

BDNF (Catalog No. DBD00, USA R&D Systems, Inc., Minneapolis, MN, USA) and TSPO (Catalog No. SEJ628Hu, CLOUD-CLONE CORP., Katy, TX, USA) were measured using an enzyme-linked immunosorbent assay (ELISA) method. The change in color was measured using a Thermo Scientific™ Multiskan™ GO microplate spectrophotometer at a wavelength of 450 nm ± 10 nm. The TSPO concentration in the samples was then determined by comparing the O.D. of the samples in the standard curve. 

The laboratory technicians who analyzed BDNF and TSPO were blinded to the group of the stroke cases and the control cases as well as the characteristics of the study participants. Both assays were performed in duplicate. The minimal detectable dosage for this kit, <0.127 ng/mL, was determined to be zero.

### 2.7. Statistical Analysis

The results were expressed as frequency and percentage for categorical variables, and as mean ± standard deviation (SD) or median (interquartile range [IQR]) for continuous variables. For continuous variables, comparisons between groups were carried out using Student’s *t*-test or the Mann–Whitney U test, as well as one-way analysis of variance (ANOVA). The percentage of patients with a given characteristic in each group was compared using the Chi-square test, or Fisher’s exact test in the case of small numbers. Logistic regressions were used to investigate the association between plasma BDNF and TSPO categories and having AIS, after adjusting the confounding factors. The odds ratio (OR) and 95% confidence interval (CI) were obtained. A receiver operating characteristic curve (ROC) analysis and an area under the ROC curve (AUC) analysis were applied. Sensitivity and a function of 1-specificity were used to measure the accuracy of BDNF or/and TSPO for discriminating AIS patients from the normal controls. ANOVA and least square difference (LSD) post hoc test was performed to test the differences in biomarker levels in 5 sampling time (in hours) categories: <24, 24–48, 48–72, 72–96, 96–120. All of the calculations were performed using IBM SPSS software for Windows, version 23. All of the reported p-values were two-sided, and the significance was accepted at *p* < 0.05.

## 3. Results

### 3.1. Baseline Characteristics of the Study Participants

**Figure 1:**
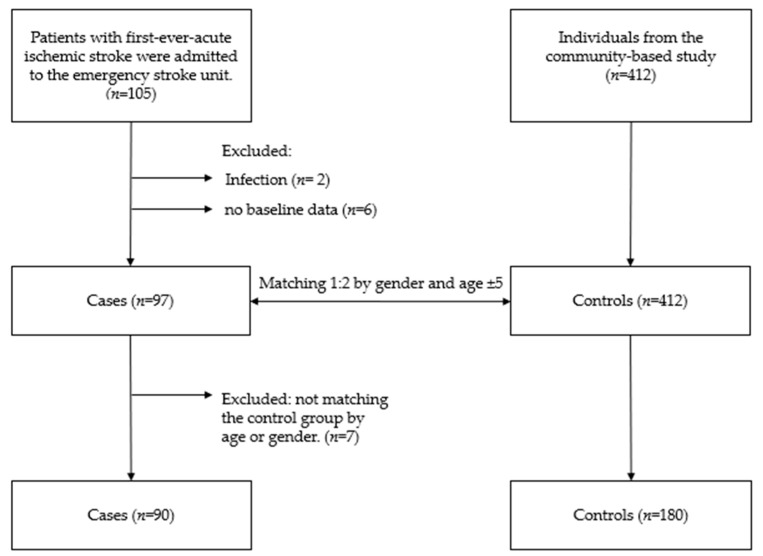
Study flow-chart.

Figure 1 shows the study flow chart. Ninety acute stroke patients (male *n* = 61 and female *n* = 29) and 180 control subjects (male *n* = 122 and female *n* = 58) were included in the study. Stroke patients had significantly higher levels of SBP (163.04 ± 41.98) and DBP (90.62 ± 26.99), and there were higher proportions of diabetes (48.9%) and hypertension (71.1%) among them. Stroke patients had significantly higher levels of WBC, BUN, creatinine, Hs-CRP, HbA1c, and glucose than the control group (Table 1). Supplementary Table S1 shows the characteristics of 90 patients with acute ischemic stroke. Twenty-one patients were treated with an intravenous tissue plasminogen activator (23.3%). According to the etiology of stroke, 28 patients were diagnosed with large artery atherosclerosis, 3.3% with small artery atherosclerosis, 12 (13.3%) with cardio-embolism, 3.3% with determined causes, and eleven (12.2%) with undetermined causes. There were 69 patients with a modified Rankin scale score of >3 or death at discharge (76.7%). Eleven patients died (12.2%), and 21 suffered a severe stroke (NIHSS > 21) (23.3%).

### 3.2. Biomarker Level

**Figure 2:**
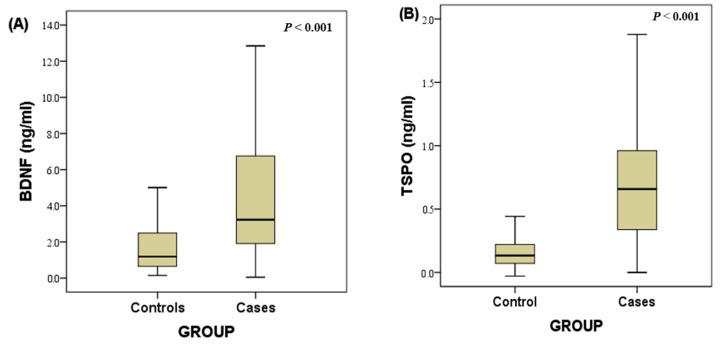
(**A**) Box plots of the plasma level of BDNF between stroke patients and individuals without acute ischemic stroke. (**B**) Box plots of the plasma level of TSPO between stroke patients and individuals without acute ischemic stroke. *p* value was obtained using a 2-sample *t* test.

Table 2 shows the levels of the biomarkers BDNF and TSPO in the stroke patients and the healthy controls. The concentration of BDNF was significantly higher in the stroke patients as compared to the control group, namely 3.227 ng/mL (IQR 1.867–6.801 ng/mL) vs. 1.187 ng/mL (IQR 0.640–2.546 ng/mL). In addition, TSPO levels significantly increased in the stroke patients compared to the control group, namely 0.658 ng/mL (IQR 0.333–0.964 ng/mL) vs. 0.133 ng/mL (IQR 0.000–0.222 ng/mL). The results are also visualized in Figure 2.

### 3.3. Sampling Time Analysis in Stroke Patients

The sampling time ranged within five days of hospital admission. We categorized the sampling time into 1–24, 25–48, 49–72, 73–96, and 96–120 h following admission, and have shown the box plot in the Supplementary Figures S1 and S2. According to the one-way ANOVA for sampling time categories, the differences in BDNF were significant (ANOVA, F(4.80) = 2.566, *p* = 0.044 in Supplementary Figure S1), but were found to be insignificant for TSPO (ANOVA, F(4.80) = 0.380, *p* = 0.822 in Supplementary Figure S2).

In Supplementary Figure S1, the BDNF level was low at <24 h of admission (average 2.94 ng/mL), though the level increased sharply at 25–48 h (average 6.79 ng/mL) and then gradually decreased to 2.77 ng/mL 96 h later. The variation of BDNF level at 25–48 h was wider than in other categories. An LSD post hoc test for multiple comparisons found that the BDNF was significantly different between 1–24 and 25–48 h (*p* = 0.020), as well as between 25–48 and 49–72 h (*p* = 0.028), and between 97–121 and 25–48 h (*p* = 0.011). In addition, for the patients who were sampled <24 h following admission, the mean value of BDNF was 2.94 ± 2.16 ng/mL, which was significantly lower than the mean in patients sampled >24 h from admission (4.90 ± 4.16 ng/mL, *p* = 0.047).

In Supplementary Figure S2, there were no significant differences in TSPO levels among the five blood sampling time categories, nor between the pairwise post hoc comparisons. 

### 3.4. ROC Analysis of Biomarkers 

The univariate analysis showed that, compared with the reference group, the likelihood of associations between BDNF levels and AIS were significantly increased in subjects with ≥1.615 ng/mL (OR, 12.46; 95% CI, 6.40–24.24). Furthermore, multivariate analysis showed that patients with higher BDNF levels were more likely to have stroke than patients with lower BDNF levels, after adjusting for age and sex (OR, 13.62; 95% CI, 6.83–27.14) as well as for comorbidities (DM, HTN, WBC, BUN, creatinine, HbA1c, HDL, and Hs-CRP), (OR, 6.77; 95% CI, 2.02–22.68). Compared with the reference group, the association between TSPO levels and AIS was significantly increased in subjects with ≥0.194 ng/mL (OR, 5.68; 95% CI, 3.19–10.09). Furthermore, multivariate analysis showed that patients with higher TSPO levels were more likely to have a stroke than patients with lower TSPO levels, after adjusting for age and sex (OR, 5.82; 95% CI, 3.26–10.38) as well as for comorbidities (DM, HTN, WBC, BUN, creatinine, HbA1c, HDL, and Hs-CRP,) (OR, 3.27; 95% CI, 1.09–9.82) (Table 3).

**Figure 3:**
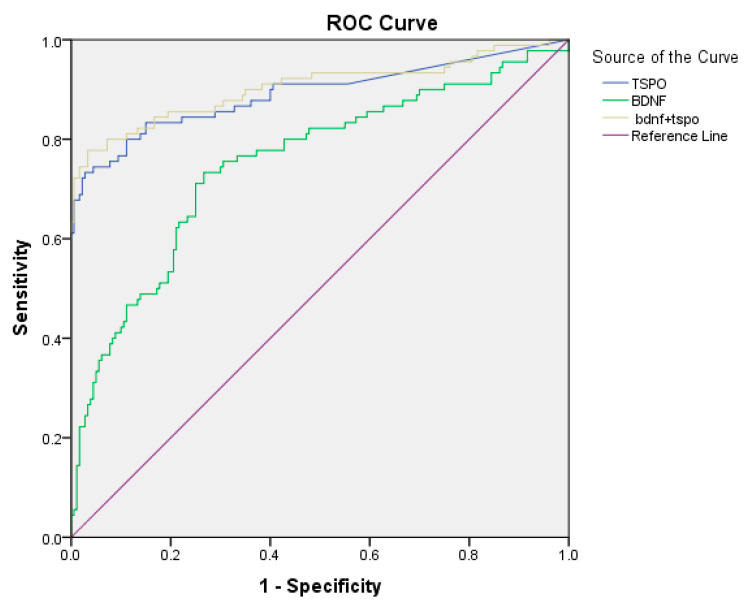
The receiver operator characteristic (ROC) curves of BDNF and TSPO for discriminating patients with AIS from individuals without acute ischemic stroke.

Figure 3 shows the receiver operator characteristic (ROC) curves, demonstrating sensitivity as a function of 1-specificity for discriminating patients with AIS from individuals without acute ischemic stroke, based on plasma BDNF levels with the AUC at 0.76 (95% CI 0.69–0.82 *p* < 0.001) with a cut-off value of plasma BDNF ≥ 2.171 ng/mL, a sensitivity of 73%, and a specificity of 73.3%. A cut-off value of plasma TSPO ≥ 0.375 ng/mL yielded a sensitivity of 73% and specificity of 97.2% with the AUC at 0.89 (95% CI 0.84–0.94 *p* < 0.001). A combination of plasma BDNF and plasma TSPO levels with the AUC at 0.90 (95% CI 0.86–0.95 *p* < 0.001) yielded a sensitivity of 78% and a specificity of 96.7% (Table 4).

## 4. Discussion

In this study, we observed that plasma BDNF and TSPO levels were significantly higher in AIS patients compared with the control group. The association was still significant after adjusting for age, gender, comorbidities (HTN, DM), and biomarkers, (BUN, creatinine, HbA1c, WBC, HDL, and Hs-CRP). Plasma TSPO levels, rather than BDNF, had a good ability to discriminate stroke patients from individuals without acute ischemic stroke (AUC = 0.89). The involvement of BDNF barely improved AUC (=0.90). In the acute and subacute phases of stroke patients, the trend of BDNF changed drastically, with TSPO being more stable than BDNF.

Recent research has focused on the effects of brain-derived neurotrophic factor, a mammalian neurotrophic factor which regulates neurogenesis, neurodegeneration, and neuroplasticity [27]. It is believed that BDNF and TSPO could be key biomarkers of acute ischemic stroke in newly admitted stroke patients within the first few hours of admission. A closer look at the mechanism of BDNF and TSPO showed that they were associated with ischemic stroke. Translocator protein (TSPO) was formerly known as a receptor of peripheral benzodiazepine, though it is currently known as a receptor present throughout the brain and the body at large. There has been a lot of evidence showing that the expression of TSPO correlates with the activation of microglia, which is highly associated with brain injuries and neuroinflammation conditions [28]. The use of TSPO as a diagnostic tool for determining the extent of neuroinflammation damage is quite appealing, due to the fact that inflammatory mediator expression levels within brain cells under physiological conditions are relatively low and rapidly increase in response to neuroinflammatory stimuli, particularly microglia, astrocytes, and macrophages [29]. Studies have revealed that there are two general phenotypes of activated microglia: the proinflammatory M1 and the alternatively activated anti-inflammatory M2 [30]. M1 microglia are pro-inflammatory and release inflammatory cytokines (TNF, IL-6, and IL-12) to facilitate injury-mediating responses, while M2 microglia are anti-inflammatory and possess neuroprotectants (IL-4 and IL-13), as well as growth factors (BDNF and IGF) [26]. Microglia activation in ischemic stroke patients is limited to 72 h. After that, the binding potential reaches its highest levels in the core infarction zone, peri-infarction zone, and contralateral hemisphere, doing so 30 days after the stroke. Potentially, neuroprotection may extend beyond traditional time windows [31].

Constitutive expression of TSPO is lower, and frequently below detection limits in a normal, healthy brain, but is higher in the majority of peripheral organs, including the adrenals, kidneys, spleen, and liver [32]. On the other hand, high levels of TSPO can be observed in the brain after damage or during actively advancing tissue disease [33,34]. Betlazar et al. found that the microglia in a normal mouse brain does not express TSPO in an easily recognizable way. They confirmed the hypothesis that TSPO is not expressed by resting glial cells, but that it is sparsely expressed at low levels in the normal brain parenchyma, where it interacts with endothelial cell mitochondria [34]. TSPO ligands, such as PK11195, Etifoxine, Emapunil, and 2-Cl-MGV-1, show the possibility of targeting TSPO for the treatment of brain diseases and disorders [26]. This study also considered the diverse and often contradictory findings of TSPO ligand uptake in various CNS illnesses; it remains one of the most promising “tools” for non-invasive diagnosis. Since TSPO expression is altered not just as a result of microglial activation but may also be a sign of the severity of disease and/or progression, it has been investigated for the diagnosis of neurological conditions.

Apart from clinical trials, several scholars have tried to establish the association between both BDNF and TSPO with AIS conditions from a genetics perspective. For example, Kim et al. [35] claimed that the high methylation status of BDNF was correlated with low neural BDNF expression, and that this was an indication that BDNF is highly associated with ischemic stroke incidence. In preliminary studies, Kim et al. [35] stated that the number of BDNF Met alleles is highly associated with the incidence of ischemic stroke. However, Zhou et al. argued that BDNF Met alleles have no association with BDNF levels in the plasma. Furthermore, several studies that explore BDNF’s role have pointed out indirectly that the alteration of plasma levels of BDNF in stroke patients tends to significantly contribute to ischemic stroke pathogenesis [18].

The intensity of the stroke and the time point after stroke onset are two potential causes of the different effects. This migration of BDNF through the BBB might be important during the postischemic phase, since BDNF can exert a protective action at the site of the lesion. These investigations did not, however, clearly define the stage of the stroke at the time the blood sample was taken. In the very acute stage (herein 24 h), increased plasma BDNF is linked to severe stroke in rats [36]. Moreover, greater levels of BDNF since admission and worse clinical outcomes were associated [37]. Another study showed the BDNF levels at 72 h were associated with a variety of clinical outcomes [37]. Recent research has revealed that BDNF is crucial for poststroke mobility and ischemia. Plasma BDNF levels are therefore prospective indicators of both short- and long-term outcomes in the first days following a stroke [24].

Meta-analysis research has revealed that stroke patients had lower levels of BDNF than healthy controls [23]. Algin et al. demonstrated that BDNF levels could discriminate between stroke patients and controls, as they were considerably lower in the stroke group than in the controls [25]. Overberg and their colleagues reported that patients with acute stroke have 2.5-fold lower plasma levels of BDNF than age- and gender-matched healthy controls. In their study, blood samples were collected within the first day following admission. Mourão, A.M., et al. reported that they assessed 50 individuals at three time points: immediately after being admitted to the hospital (i.e., within 24 h of their stroke), 72 h after admission, and right before being discharged. Although there was some variation, the plasma levels of BDNF in patients with ischemic stroke did not fluctuate significantly during their hospital stay in their study [37]. However, a small number of studies have also demonstrated that the amount of BDNF rises in stroke patients, compared to that of healthy controls [22,38]. These researchers showed the variate BDNF levels associated with complicated patient enrollment and following clinical outcomes in stroke patients.

In our observation of stroke patients, the BDNF level was low at <24 h of admission (average 2.94 ng/mL). This level increased sharply at 25–48 h (average 6.79 ng/mL), and then gradually decreased to 2.77 ng/mL at 96 h later. The variation of BDNF level at 25–48 h was wider than it was in other categories. This indicated that heterogeneity in some factors (such as the stroke severity, therapy, or personal characteristics) was an underlying cause, rather than sample size, given that group 25–48 had the largest sample size. These findings were similar to several studies, including Chen’s. According to Chan et al., stroke patients (*n* = 75) had slightly higher blood levels of BDNF compared to healthy controls (*n* = 56). When these subgroups were compared to the controls, there were no statistically significant differences in the results for BDNF levels at one day, one week, nor three weeks following the stroke [22]. Individuals younger than 65 years had much higher levels of BDNF in the first 24 h after a stroke [38]. In addition, our study also showed the BDNF level decreased to lower levels four days following admission, which was the common time point for collecting blood samples from patients. In the same time frame, TSPO was more stable, keeping higher for several days in stroke patients, and improving the diagnostic capability.

In a preclinical study, Schabtz et al. [39] claimed that BDNF can induce an antiapoptotic mechanism after insults caused by stroke, as well as impede the secondary death of neural cells. In addition, conditional BDNF knockout mice showed high levels of depression-related behaviors, thus demonstrating that low BDNF expression might lead to depressive disorders [39]. In the same vein, Chen et al. [40] confirmed that low concentrations of BDNF could lead to a low occurrence of neurogenesis in the hippocampus, thereby potentially inducing a stroke. Therefore, the aforementioned pieces of empirical research undoubtedly support the idea that low levels of BDNF significantly contribute to depression pathophysiology. This study strongly contributes to the discussion of BDNF’s applicability as a biomarker for the early screening of AIS patients. Ideally, it is clear that the precise nature of the association between changes in levels of BDNF and ischemic stroke pathogenesis should be determined; thus, more detailed studies are required to explore circulating BDNF’s role in the development of neurodegenerative diseases.

Following the results of our study and the review of the existing literature, it is evident that plasma TSPO levels are significantly higher in AIS patients compared with control groups, and they can therefore serve as a biomarker of ischemic stroke in the first few hours of stroke onset. Although studies have reported that TSPO expression depends on the time from stroke onset, the levels return to low values when nerve healing is completed. However, in the acute and subacute phases of ischemic stroke, it is TSPO rather than BDNF that is more stable and available to identify or detect stroke onset. Based on the ROC curve, BDNF had little (if any) additional value to TSPO. Currently, the diagnosis of IS is mostly based on clinical examination, with confirmation being undertaken using computed tomography (CT) or magnetic resonance imaging (MRI) tests as the required approach to distinguish IS from HS. Even though a cerebral CT scan may effectively distinguish between IS and HS, only around 85% of IS is correctly identified using a CT scan within six hours of the beginning of the stroke. Although MRI is more expensive, less accessible, time-consuming, and sometimes impossible in severely ill or restless patients, it can detect acute ischemia better than CT, making it an excellent method for diagnosing acute ischemic stroke (AIS) [41,42,43]. A thrombolytic drug called tissue plasminogen activator (tPA) is used to dissolve clots, while anticoagulants like aspirin are used to stop additional ischemia. The most effective treatment for stroke patients is frequently tPA, although it must be given within the first three hours, or maybe even up to 4.5 h [44,45].

This study has several limitations. Firstly, the relatively small sample size may limit the generalization of the results. We had a limited number of cases that we were able to recruit during the study period. Secondly, blood samples were collected only once after IS symptom onset. Some patients were examined within the first few hours, whilst others were examined late. The sampling time varied according to the clinical judgment of the suspected stroke from a physician. Thirdly, we only recruited AIS patients and controls. The clinical problem was not only whether an IS can be distinguished from the control, but rather extends to whether an IS can be distinguished from other stroke types and stroke mimics, since these can also influence the biomarkers studied, such as hemorrhagic stroke, seizure, migraine, syncope, or hypoglycemia [46].

## 5. Conclusions

In conclusion, our study found that TSPO was better than BDNF at distinguishing AIS patients from controls. Increased TSPO level may be considered as a potential diagnostic biomarker in patients with acute ischemic stroke upon admission. BDNF added little improvement to the diagnostic ability of detecting stroke onset. For this reason, medical practitioners can consider adopting the measurement of BDNF and TSPO in the blood as significant biomarkers for acute ischemic stroke. However, a detailed cross-validation study with a larger sample size and diverse populations should be replicated, so as to justify the findings for proper medical practice. Future research could explore this potentially valuable avenue.

## Figures and Tables

**Table 1:** Baseline demographic and clinical characteristics of all of the study participants (both stroke patients and controls).

Characteristics	Cases (*n* = 90) N (%)/M ± SD	Control (*n* = 180) N (%)/M ± SD	*p*-Value
Age	67.7 ± 12.88	66.63 ± 11.25	0.484
Gender (Male)	61 (67.8)	122 (67.8)	1.0
Systolic blood pressure	163.04 ± 41.98	124.63 ± 17.62	<0.001
Diastolic blood pressure	90.62 ± 26.99	73.4 ± 8.99	<0.001
Hypertension	64 (71.1%)	33 (18.3%)	<0.001
Diabetes mellitus	44 (48.9%)	30 (16.7%)	<0.001
Hyperlipidemia	37 (41.1%)	85 (47.2%)	0.366
Platelet (10^3^/uL)	236.76 ± 99.47	229.18 ± 60.94	0.437
White blood cell (10^3^/uL)	10.3 ± 4.33	5.85 ± 1.4	<0.001
Hematocrit (%)	41.80 ± 5.66	42.03 ± 3.56	0.683
Blood urea nitrogen (mg/dL)	20.31 ± 9.57	17.0 ± 6.1	<0.001
Hs C-reactive protein (mg/dL)	5.72 ± 6.41	0.22 ± 0.63	<0.001
Creatinine (mg/dL)	1.37 ± 1.31	0.87 ± 0.24	<0.001
Hemoglobin A1c (%)	6.59 ± 1.68	6.0 ± 0.87	<0.001
Glucose (mg/dL)	127.53 ± 47.59	96.24 ± 29.74	<0.001
Triglycerides (mg/dL)	124.05 ± 67.55	123.15 ± 141.94	0.955
Total cholesterol (mg/dL)	178.55 ± 46.46	183.16 ± 33.59	0.353
High-density lipoprotein cholesterol (mg/dL)	45.39 ± 10.79	50.54 ± 12.72	<0.001
Low-density lipoprotein cholesterol (mg/dL)	112.79 ± 36.51	113.0 ± 29.0	0.960

*p*-values refer to a two-sample *t*-test, Chi-square test, or Fisher’s exact test. M ± SD: Mean ± standard deviation.

**Table 2:** Plasma BDNF and TSPO concentration in the stroke patients and the controls.

Biomarker	All Median (IQR)	Cases Median (IQR)	Control Median (IQR)	*p*-Value
BDNF (ng/mL)	1.615 (0.8339–3.705)	3.227 (1.867–6.801)	1.187 (0.640–2.546)	<0.001
TSPO (ng/mL)	0.194 (0.091–0.417)	0.658 (0.333–0.964)	0.133 (0.071–0.222)	<0.001

**Table 3:** Logistic regression in BDNF and TSPO biomarkers with the onset risk of ischemic stroke.

Biomarkers (ng/mL)	Crude Model OR (95%CI)	Model 1 OR (95%CI)	Model 2 OR (95%CI)
BDNF (ng/mL) (≥1.615 vs. <1.615) *	12.46(6.40–24.24)	13.62 (6.83–27.14)	6.77 (2.02–22.68)
TSPO (ng/mL) (≥0.194 vs. <0.194) *	5.68 (3.19–10.09)	5.82 (3.26–10.38)	3.27 (1.09–9.82)

* Cut-off at median values. Model 1: adjusted for age and gender. Model 2: adjusted for age, gender, DM, HTN, WBC, BUN, creatinine, HbA1c, HDL, and Hs-CRP/OR (odds ratio), CI (confidence interval), DM (diabetes mellitus), HTN (hypertension), WBC (white blood cell), BUN (blood urea nitrogen), Hs-CRP (high-sensitivity C-reactive protein), HbA1c (hemoglobin A1c), and HDL (high-density lipoprotein cholesterol).

**Table 4:** Results of the receiver operator characteristic (ROC) curves of BDNF and TSPO for discriminating patients with AIS from individuals without acute ischemic stroke.

Biomarkers	AUC	Sensitivity	Specificity	PLR	Cut-Off	Y.I.	*p*-Value
BDNF	0.76	73%	73.3%	2.70	2.171	0.467	0.001
TSPO	0.89	73.3%	97.2%	26.28	0.375	0.706	0.001
BDNF + TSPO	0.90	78%	96.7%	23.64	0.428	0.744	0.001

AUC: the area under the curve; PLR: positive likelihood ratio; Y.I.: Youden index.

## Data Availability

The data presented in this study are available from the corresponding author upon request. The data are not publicly available due to privacy restrictions.

## Supplementary Materials

The following supporting information can be downloaded at: https://www.mdpi.com/article/10.3390/diagnostics13132298/s1, Figure S1. Boxplots of plasma BDNF level and the sampling time categories in acute ischemic stroke patients; Figure S2. Boxplots of plasma TSPO level and the sampling time categories in acute ischemic stroke patients; Table S1. Characteristics of acute ischemic stroke patients.
