# Supplementary material for: Brain-Derived Neurotrophic Factor (BDNF) and Translocator Protein (TSPO) as Diagnostic Biomarkers for Acute Ischemic Stroke
Source: Diagnostics (Basel). 2023 Jul 6;13(13):2298. doi: 10.3390/diagnostics13132298 (PMC10340661; doi:10.3390/diagnostics13132298)
Supplement: Supplementary file 1 [file diagnostics-13-02298-s001.zip › diagnostics-2385599-supplementary.pdf]

Supplementary table and figure

Table S1. Characteristics of acute ischemic stroke patients

	**(n=90) N**	**percentage (%)**
Current Smoker	19	21.1
Aspirin	60	66.7
t-PA treatment	21	23.3
Atrial Fibrillation	32	35.6
NIHSS		
Moderate stroke (5-15)	46	51.1
Moderate to severe stroke (16-20)	14	15.6
Severe stroke (≥21)	21	23.3
Modified Rankin scale (>3)	69	76.7
Death	11	12.2
TOAST classification		
Large Artery Atherosclerosis	28	31.1
Small artery Atherosclerosis	3	3.3
Cardioembolism	12	13.3
Determined cause	3	3.3
Undetermined cause	11	12.2

t-PA- tissue Plasminogen Activator; NIHSS- National Institutes of Health Stroke Scale

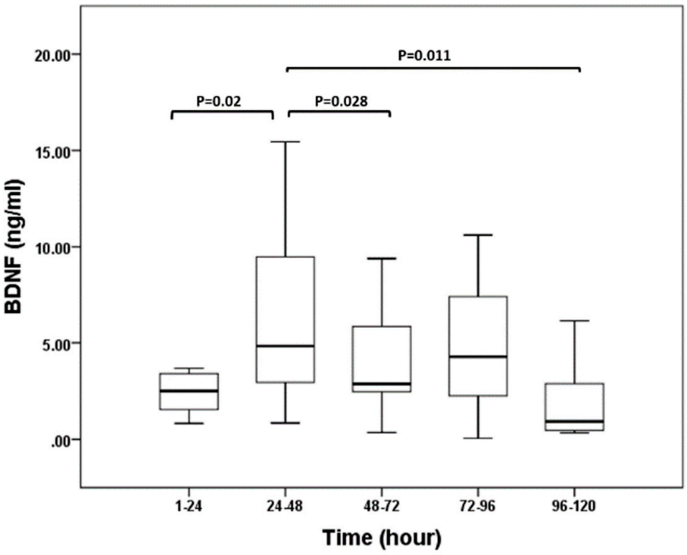

Figure S1. Boxplots of plasma BDNF level and the sampling time categories in acute ischemic stroke patients.

Footnote: 5 missing data of blood sampling time in hours were excluded. One-way ANOVA with sampling time was used (p=0.044). Least square difference in hours for multiple comparisons: 1-24 versus 25-48 (p = 0.020), 25-48 versus 49-72 (p = 0.028), and 97-120 versus 25-48 (p = 0.011). others were non-significance.

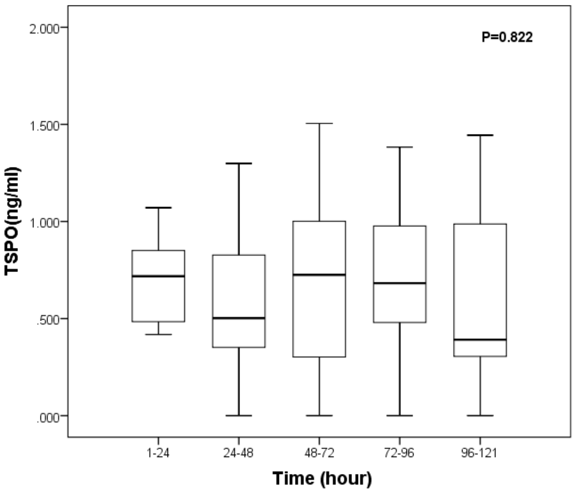

Figure S2. Boxplots of plasma TSPO level and the sampling time categories in acute ischemic stroke patients.

Footnote: 5 missing data of blood sampling time in hours were excluded. One-way ANOVA with sampling time was used (P=0.822). all post hoc comparisons were non-significant.
